# FSH regulates RA signaling to commit spermatogonia into differentiation pathway and meiosis

**DOI:** 10.1186/s12958-020-00686-w

**Published:** 2021-01-07

**Authors:** Maryam Khanehzad, Roya Abbaszadeh, Marzieh Holakuyee, Mohammad Hossein Modarressi, Seyed Mehdi Nourashrafeddin

**Affiliations:** 1 Department of Anatomy, Faculty of Medicine, Tehran University of Medical Sciences, Tehran, Iran; 2 Department of Molecular Medicine, School of Advanced Technologies in Medicine, Tehran University of Medical Sciences, Tehran, Iran; 3 Department of Biology, Tarbiat Modares University, Tehran, Iran; 4 Department of Medical Genetics, Faculty of Medicine, Tehran University of Medicine Science, Tehran, Iran; 5 Department of Obstetrics, Gynecology and Reproductive Sciences, School of Medicine, University of Pittsburgh, Pittsburgh, USA; 6 School of Advanced Technologies in Medicine, Tehran University of Medical Sciences, Tehran, Iran

**Keywords:** FSH, Retinoic acid, Spermatogonia, Spermatogenesis, Differentiation

## Abstract

**Background:**

Spermatogenesis is a complex process that is controlled by interactions between germ cells and somatic cells. The commitment of undifferentiated spermatogonia to differentiating spermatogonia and normal spermatogenesis requires the action of gonadotropins. Additionally, numerous studies revealed the role of retinoic acid signaling in induction of germ cell differentiation and meiosis entry.

**Main text:**

Recent studies have shown that expression of several RA signaling molecules including Rdh10, Aldh1a2, Crabp1/2 are influenced by changes in gonadotropin levels. Components of signaling pathways that are regulated by FSH signaling such as GDNF, Sohlh1/2, c-Kit, DMRT, BMP4 and NRGs along with transcription factors that are important for proliferation and differentiation of spermatogonia are also affected by retinoic acid signaling.

**Conclusion:**

According to all studies that demonstrate the interface between FSH and RA signaling, we suggest that RA may trigger spermatogonia differentiation and initiation of meiosis through regulation by FSH signaling in testis. Therefore, to the best of our knowledge, this is the first time that the correlation between FSH and RA signaling in spermatogenesis is highlighted.

## Introduction

Spermatogenesis is a continuous highly regulated process of male germ cell proliferation and differentiation. This process takes place in the seminiferous tubules of the testis in order for the generation of sperm throughout the lifetime. Differentiation of spermatogonia occurs through a linear process including mitotic expansions, meiotic reduction divisions, and morphological transformations. The commitment of spermatogonia to differentiation pathway happens when undifferentiated spermatogonia undergo an irreversible transition (in mouse) or division (in primate) to produce differentiating spermatogonia.

In mammals, spermatogenesis is initiated in postnatal life at puberty with differentiation of undifferentiated spermatogonia and their meiotic entry [1, 2]. Spermatogonia are able to not only self-renew and proliferate to maintain stem cell populations but also generate progenitor cells that enter the spermatogenesis process to form mature sperms. The fate of the spermatogonia population is determined by complex interactions between the germ cells, the testicular somatic cells, and different types of growth factors.

**Fig. 1:**
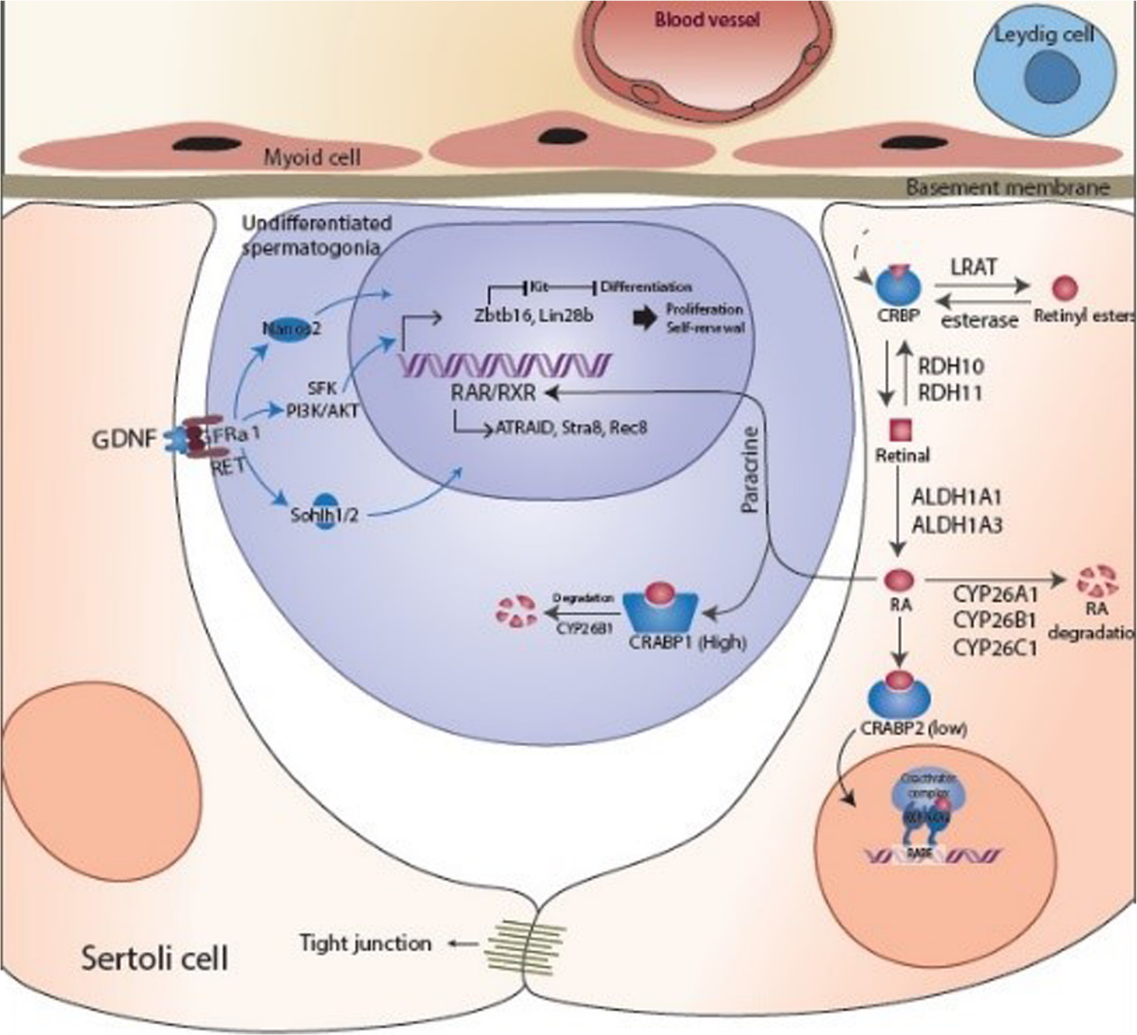
The possible paracrine mechanisms controlling self-renewal of spermatogonia in juvenile testis. Spermatogonia are in close contact with Sertoli cells which provide structural support and paracrine factors to the developing spermatogenic cells. The self-renewal and proliferation factor GDNF expressed by Sertoli cells is responsible for transcriptional activation of Zbtb16 and Lin28B and thereby preventing spermatogonia to enter differentiation pathway. Sertoli cells are the source of paracrine RA signaling pathway. In Sertoli cells, retinol is oxidized to retinal by RDH10 and then to RA by RALDH1a1. However, the amount of RA produced by Sertoli cells within juvenile testis is not robust to initiate spermatogonia differentiation possibly due to the low amount of CRABP2 which is considered for activating of RA signaling in Sertoli cells as well as the high amount of CRABP which is considered for degrading of RA signaling within spermatogonia. Black arrows refer to retinol metabolism and RA signaling pathway. Blue arrows refer to endogenous factors that promote spermatogonia proliferation and self-renewal but at the same time inhibits spermatogonial differentiation and meiotic entry. RBP4, retinol binding protein 4; STRA6, stimulated by retinoic acid gene 6 cell membrane receptor; CRBP, cellular retinol binding protein; LRAT, lecithin retinol transferase; RDH10, retinol dehydrogenase 10; RALDH1a1, retinaldehyde dehydrogenase 1a1; RA, retinoic acid; CYP26, cytochrome *P*-450 enzymes from the cyp26 family; CRABP, cellular retinoic acid binding protein; RAR, retinoic acid receptor; RXR, rexinoid receptor;. RARE, retinoic acid respond element; ATRAID, All-Trans Retinoic Acid Induced Differentiation Factor; Stra8, stimulated by retinoic acid 8; Rec8, REC8 like meiotic recombination protein; GDNF, Glial cell-derived neurotrophic factor; GFRα1, GDNF family receptor α1; Nanos2, Nanos Homolog 2; Sohlh1/2, Spermatogenesis- And Oogenesis-Specific Basic Helix-Loop-Helix-Containing Protein 1,2; Zbtb16, Zinc finger and BTB domain containing 16; Lin28B, Lin-28 homolog B; c-Kit, V-Kit Hardy-Zuckerman 4 Feline Sarcoma Viral Oncogene-Like Protein

The commitment of undiferrentiated spermatogomia to differentiating spermatogonia and normal spermatogenesis requires the action of gonadotropins. Secretion of gonadotropins including luteinizing hormone (LH), and follicle-stimulating hormone (FSH) by the pituitary gland is under the control of the hypothalamic gonadotropin-releasing hormone (GnRH). Spermatogenesis is regulated by signals from gonadotropins’ effects on the Leydig and Sertoli cells which express luteinizing hormone receptor (LHR) and follicle stimulating hormone receptor (FSHR), respectively. In the absence of FSH and LH only undifferentiated germ cells and Sertoli cells are present in the seminiferous tubules of the testis [3]. The prepubertal or juvenile phase of testis development is characterized by a protracted hypogonadotropic state in which the epithelium of seminiferous tubules consists merely of Sertoli cells and undifferentiated germ cells [4]. All germ cells in the premature or juvenile testis are undifferentiated spermatogonia, which are proliferating in a relatively gonadotropin-independent manner (Fig. 1) [5, 6].

Since 1925, it has been demonstrated that vitamin A is required for normal spermatogenesis [7], and vitamin A deficient (VAD) animals are infertile [8]. Vitamin A deficiency leads to the spermatogenesis arrest at early meiotic stages [9]. In VAD animals only Sertoli cells and undifferentiated spermatogonia are present in the seminiferous tubules of the testis [10]. However, the differentiation block in VAD animals can be removed by retinol [10].

A large number of recent studies, mainly in rodents, have revealed that retinoic acid (RA), a biologically active form of vitamin A, plays an essential role in the spermatogenesis process. RA is considered to be responsible for the cyclic differentiation of germ cells in the adult testis and the continual production of sperm. It has been found that RA is critical for the induction of germ cell differentiation, initiation of meiosis and normal spermiogenesis [11–13]. RA is needed for the transition of spermatogomia A to A1 and meiotic initiation [14, 15], and only undifferentiated spermatogonia and Sertoli cells exist within the seminiferous epithelium of the VAD murine testes [16, 17]. In mice, the commitment of undifferentiated spermatogonia to meiosis occurs during stages VII to IX of the cycle of the seminiferous epithelium via the RA pulse. It has been suggested that RA may activate adjacent target cells in a paracrine manner [18]. In the murine testis, it has been stated that Sertoli cells might be RA generating cells and the A spermatogonia might be RA responsive cells. If RA, as an extrinsic factor, contributes to controlling the cycle of the seminiferous epithelium in the testis, the question is which cells, enzymes, and receptors are responsible for generating, responding to, and degrading the RA pulse.

It has been proposed that gonadotropins may trigger the differentiation of spermatogonia and their meiotic entry through regulation of RA signaling within the seminiferous epithelium in the testis. This raises a new hypothesis about the mechanisms of gonadotropins to control spermatogenesis via RA [19]. Recent study by Plant’s lab has revealed that the expression of some RA signaling molecules including All-trans-retinol dehydrogenase 7 (ADH7), All-trans retinoic acid-induced differentiation factor (ATRAID), Retinol dehydrogenase 10 (RDH10) and Cellular retinoic acid-binding protein 2 (CRABP2), were significantly changed after LH and FSH stimulation for 48 and 96 h [20]. On the other hand, our recent study demonstrated that CRABP1 and CRABP2 were down and up-regulated, respectively; after LH and FSH stimulation for 11 days [21]. The purpose of this article is to summarize a developmental perspective on the knowledge about the possible correlation between gonadotropins and Retinoic acid signaling to commit undifferentiated progenitor spermatogonia to the entry into differentiation pathway and meiosis.

### Development of undifferentiated spermatogonia

In mice, the formation of the somatic and germ cell lineage occurs independently within the testis. During embryonic development, the bipotential gonads cells are committed to become the Sertoli cells of the testis. The absence of Sex-determining region Y protein (Sry) expression leads to the formation of granulosa cells in the developing ovary in females. Primordial germ cells (PGCs) appear in the epiblast and migrate to the developing gonadal ridge [22]. The interaction between PGCs and embryonic Sertoli cells leads to the formation of embryonic seminiferous cords [23]. Then PGCs undergo a mitotic proliferation to become gonocytes or prospermatogonia. In the juvenile testis, prospermatogonia are located in the middle of the seminiferous cords and after several transitions migrate to the periphery to form spermatogonia.

In the first round of spermatogenesis in murine testis, a unique neurogenin 3 (NGN3) negative pool of prospermatogonia transforms into A1 spermatogonia while the NGN3 positive spermatogonia remain undifferentiated stem/progenitor cells. The NGN3 negative A1 spermatogonia are observed at 2 dpp, and then differentiate into A2, A3, A4 and type B spermatogonia at 5 dpp [24]. Subsequent rounds of spermatogenesis arise from mitotic expansion of stem/progenitor cells, which are considered as “undifferentiated” spermatogonia. The mitotic divisions of undifferentiated spermatogonia allow continuous production of more undifferentiated germ cells defined as A paired spermatogonia (Apr). The Apr cells then divide to form A aligned (Aal) spermatogonia. The undifferentiated Apr and Aal cells are considered as transit amplifying progenitor germ cells, which further differentiate into A1 spermatogonia that are committed to differentiation pathway and meiotic entry [25]. In mice, Aal transforms into A1 differentiating spermatogonia without cell division. The transition of Aal spermatogonia to A1 appears to be irreversible [26]. Transformation of Aal to A1 spermatogonia is followed by six cell divisions to produce A2, A3, A4, In, and B spermatogonia, and preleptotene spermatocytes [26].

### Endocrine control of spermatogenesis (hypothalamus-pituitary-testis axis)

Normal spermatogenesis in mammals requires neuroendocrine activity along the hypothalamic-pituitary-testicular axis. In rodents, the first wave of spermatogenesis and spermatogonial differentiation occurs immediately after birth [27]. Whereas, higher primates display three different phases of postnatal activity before adulthood including neonatal, juvenile and puberty. The neonatal phase exhibits hypothalamic-pituitary-testis axis activity like adult but without spermatogenesis. During juvenile phase, the hypothalamic-pituitary- testis axis is quiescent. In the pubertal phase, the hypothalamic-pituitary-testicular axis activity is reinitiated and spermatogenesis begins [5].

Gonadotropin-releasing hormone (GnRH) is a central hypothalamic signal to the pituitary gland that expresses GnRH receptors. Two distinct gonadotropins, follicle-stimulating hormone (FSH) and luteinizing hormone (LH), are released in a pulsatile fashion in response to pulsatile release of GnRH [28, 29]. In the testis, two specific transmembrane receptors, FSHR and LHR mediate the actions of gonadotropins, FSH and LH, respectively. Within the testis, FSHR is selectively expressed by Sertoli cells in the seminiferous tubules; whereas, LHR is expressed by Leydig cells in the interstitial space [30]. Therefore. FSH directly, but LH indirectly via androgen receptor (AR), acts on spermatogenesis through the regulation of Sertoli cell factors. In response to LH signaling, testosterone is produced by the Leydig cells in a pulsatile fashion, and in response to FSH, Inhibin-α, a non-steroidal hormone, is produced by the Sertoli cells in a non-pulsatile manner [31]. The gonadal hormones, testosterone and inhibin, act as feedback signals to maintain the physiological action of the hypothalamic-pituitary axis. The major role of gonadotropins FSH and LH is to establish the normal spermatogenesis and sperm production during puberty and adulthood.

Studies in animal models of rodents such as the hypogonadal mouse and the hypophysectomized rat have shown that FSH increases the number of spermatogonia and spermatocytes; however, FSH was unable to generate spermatids in the absence of androgen [32–34]. In men and nonhuman primates, FSH acts through the Sertoli cell to facilitate the proliferation and self-renewal of spermatogonia and their transition into spermatocytes. FSH deficient monkeys inhibit spermatogonial proliferation and their transition to spermatocytes [35]. Moreover, the number of spermatogonia was increased after FSH stimulation [36]. On the other hand, FSH treatment of prepubertal monkeys initiated spermatogenesis and increased the number of Sertoli cells and resulted in the appearance of differentiating B spermatogonia and spermatocytes in the testis [37]. FSH is essential to maintain fertility in men. Puberty did not occur in men with loss of function mutations in FSH receptor [38]. Studies of men with FSH deficiency of unknown etiology demonstrated that FSH is associated with initiation of spermatogenesis and fertility [39–41].

### Cycle of the seminiferous epithelium

The cycle of the seminiferous epithelium is defined as the continuous commitment of undifferentiated spermatogonia to meiosis and formation of elongated spermatids [42]. The wave of the seminiferous epithelium was defined by Clermont as a complete series of the successive cell associations found along a seminiferous tubule in the testis [42, 43].. In the murine testis**,** the initiation of the spermatogenic process occurs every 8.6 days and the duration of A1 spermatogonia development to the point of spermiation takes 35 days [43]. The cellular associations of spermatogonia differentiation give rise to 12 stages of the cycle of the seminiferous epithelium. Prior to the differentiation of spermatogonia in the murine testis, the undifferentiated A spermatogonia proliferates randomly during stages X-II of the cycle of the seminiferous epithelium. The division of A1 to B spermatogonia is coordinated during stages X to VI of the cycle. Commitment of A spermatogonia to the differentiation pathway is a rapid transition at stage VII to IX of the cycle of the seminiferous epithelium [25]. In primate, two types of undifferentiated spermatogonia, A-dark and A-pale, are present in the testis. In rhesus monkey, the cycle of the seminiferous epithelium is composed of 12 distinct stages, and differentiation of undifferentiated Ap spermatogonia to differentiating B1 spermatogonia occurs during a rapid division at stage IX of the cycle of the seminiferous epithelium [25].

### Control of spermatogenesis: role of RA signaling

Retinoic acid (RA) activity in the testis leads to the generation of the cycle of the seminiferous epithelium and normal spermatogenic wave [7]. Recent studies have revealed that RA acts in a pulsatile manner and periodically drives spermatogonial differentiation and meiotic onset. Here we discuss the role of RA signaling molecules involved in the cycle of the seminiferous epithelium and spermatogenesis.

#### Retinoic acid receptor

Vitamin A in the form of retinol in blood is transported to testis tissues via serum retinoid binding protein 4 (RBP4). Stimulated by retinoic acid 6 (Stra6), a plasma membrane protein, is the receptor for RBP4/retinol complex in the testis. Stra6 is thought to mediate the uptake of retinol from blood into cells. Stra6 is expressed only on the basal membrane surface of Sertoli cells in the adult mouse testis in a stage specific manner during stages VIII-IX [44, 45]. The comprehensive expression analysis of genes involved in RA signaling in juvenile monkey testes has revealed that the expression of STRA6 increases in testes treated with gonadotropins after 11 days [21]. However, it has been shown that Stra6 is not essential for maintaining vitamin A homeostasis in testis tissue, and Stra6-deficient mice are fertile [45].

#### Retinol storage

Vitamin A (retinol) is important for reproduction and post-natal life development [46]. Retinol (ROH) is carried in the blood circulation as retinyl esters bound to retinol-binding protein (RBP), a carrier protein encoded by the *Rbp4* gene [47]. FSH promotes the retinoic acid biosynthesis from retinol and also the storage of retinol esters in Sertoli cells [48]. In granulosa cells, FSH induces differentiation and follicular development through increased retinol uptake from serum and RA biosynthesis [49]. A recent gene expression analysis using microarray has shown that retinol binding protein 4 (*RBP4*) would be down-regulated during the onset of puberty in fish [50].

#### Retinol dehydrogenases

The first oxidation step of Retinol to RA is controlled by Retinol dehydrogenase 10 (RDH10) [51]. Studies of RDH10 null mice have demonstrated that RDH10 is critical for RA signaling during embryogenesis [52–54]. Testis has an intrinsic ability to produce RA [55, 56]. Both Sertoli and germ cells contain RDH10 to oxidize retinol to retinal [57]. Sertoli cells-specific deletion of Rdh10 in postnatal mouse testes causes a mild spermatogenesis arrest at the Aal to A1 transition [57], while simultaneous Rdh10 deletion in both Sertoli and germ cells contributes to a complete RA deficiency and, as a result, differentiation of spermatogonia does not occur and only undifferentiated spermatogonia is found in the testis. However, spermatogenesis would recover after 4 weeks without administration of exogenous RA [57]. Gene deletion experiments conducted by Raverdeau et al., revealed that the pachytene spermatocytes are the probable source of RA in the recovered mice as a result of incomplete gene deletion [58]. RDH11, another member of the short chain dehydrogenase/reductase family, is localized in pachytene spermatocytes of the murine testis across the spermatogenic stages [59]. Recent study has revealed that the expression of RDH10 was up-regulated after 48 and 96 h of LH and FSH stimulation of juvenile rhesus monkey testis [20]. These findings show that RDH10 may play an important role in RA biogenesis and signaling in the monkey testis and it is involved inevitably in the development of male germ cells and spermatogenesis.

#### Retinaldehyde dehydrogenases

A previous comprehensive study has revealed that three retinaldehyde dehydrogenase enzymes, ALDH1A1, ALDH1A2, and ALDH1A3, are expressed in the mouse testis [60]. In the developing and adult mouse testes, *Aldh1a1* and *Aldh1a3* transcripts are expressed in Leydig and Sertoli cells [60]. Whereas, *Aldh1a2* transcripts are localized in a stage specific manner (VII–XI) in pachytene spermatocytes after postnatal day 20 [60]. Based on the abundance and specific localization of *Aldh1a1* in Sertoli cells, it has been suggested that ALDH1A1 is the main source of RA within the seminiferous epithelium [61]. Unique cellular localizations of *Aldh1a1* and *Aldh1a2* in the intratestis tissue implies that they may have specific roles in RA formation [61]. Deletions of all three retinaldehyde dehydrogenase in Sertoli cells resulted in a block of spermatogenesis at the A to A1 transition [58]. Consequently, the paracrine action of RA from Sertoli cells on germ cells is essential to initiate the A to A1 transition [58]. A recent study in Plant’s lab has shown that *ALDH1A1* protein is expressed in Sertoli cells of seminiferous epithelium in the juvenile and adult monkey testis (unpublished data). Altogether, ALDH1A1 is a contributing factor in the biogenesis of RA in Sertoli cells. Therefore, ALDH1A1 plays direct roles in the development of Sertoli cells and thereby provides paracrine stimulations involved in the spermatogenesis process of the monkey testis.

It has been shown that the expression of genes involved in FSH-induced follicular development was impaired after inhibiting ALDH activity by a specific inhibitor, and apoptosis significantly was increased in the granulosa cells [62]. It has been found that trichostatin A (TSA), a selective inhibitor of histone deacetylase in mammals, significantly has increased gene expression of the FSHβ subunit as well as *Aldh1a1* [63]. A recent study by Kawai et al., has revealed that the expression of *Aldh1* isoenzymes such as *Aldh1a1* and *Aldh1a7* is significantly increased within mice ovaries after FSH treatment [49].

It has been suggested that higher serum gonadotropins, LH and FSH, significantly are associated with lower levels of ALDH1A2 protein in the testis [64]. ALDH1A2 protein was detected in undifferentiated spermatogonia, spermatocytes, and spermatids in the human testis [61]. A recent study has investigated the expression of RA-metabolizing enzymes during post-natal testicular development in dogs and revealed that ALDH1A2 mRNA level in peripubertal testis was greater than in the adult testes [65]. Our recent study has shown that the expression of *ALDH1A2* mRNA is down-regulated in the adult monkey testis after treatment with gonadotropins for 11 days [21]. Altogether, ALDH1A2 is the main enzyme involved in RA biosynthesis in human germ cells, and relevant protein levels correlate with the number of germ cells and male infertility [64].

#### Cytochrome P-450 enzymes

RA is inactivated by three forms of cytochrome P-450 enzymes including cytochrome P450, family 26, subfamily a, polypeptide 1 (CYP26A1); cytochrome P450, family 26, subfamily b, polypeptide 1 (CYP26B1); and cytochrome P450, family 26, subfamily c, polypeptide 1 (CYP26C1) [18, 66]. Degradation of RA is critical for regulation of RA concentrations within testis and normal spermatogonial differentiation. The balance between RA synthesis by retinaldehyde dehydrogenase enzymes and oxidative degradation of RA by cytochrome P450 enzymes controls RA concentrations in tissues. It has been suggested that the expression of the RA metabolizing enzyme Cyp26b1 in the immature testis shields germ cells from the meiosis-inducing effect of RA [11, 67]. In the embryonic mouse testis, the expression of CYP26B1 in Sertoli cells is responsible for RA degradation and thereby prevents the immature germ cells from entering meiosis [11, 12, 68]. Cyp26b1 gene deletion on the embryonic mouse testis shows immature meiosis entry and germ cell apoptosis [67, 69].

In the postnatal and adult mouse testis, the expression of Cyp26b1 is restricted to the peritubular myoid cells and it insulates seminiferous epithelium from the extratubular source of RA. Hence, cells within the seminiferous epithelium are responsible for synthesizing and controlling RA concentrations [60]. Interestingly, in the neonatal mouse testis, CYP26B1 protein was detected in a heterogeneous pattern in germ cells via immunohistochemistry [70]. Furthermore, treating testes with a CYP26 enzyme inhibitor causes an increase in the number of germ cells expressing STRA8 [70]. By eliminating CYP26A1 and CYP26B1 singly or combined in either germ cells or Sertoli cells, the subfertility phenotype is generated [71]. A recent gene expression analysis using microarray in fish showed that the expression of *Cyp26a1* was down-regulated during the onset of puberty [50]. Our recent study has demonestrated that CYP26B1 protein was detected in the cytoplasm of undifferentiated spermatogonia in developing monkey testis and thereby may prevent their differentiation and entry to meiosis [72]. Interestingly, the pattern of CYP26B1 protein expression has been stage specific along seminiferous epithelium in the adult monkey testis, with highest expression level in early meiotic germ cells within seminiferous epithelial stages X-XII and lowest expression level in stages VII-IX of the seminiferous epithelium where undifferentiated Type A spermatogonia divides and forms differentiating Type B spermatogonia and enters meiosis. These findings, therefore, lead us to suggest that stage-specific expression of CYP26B1 in the adult monkey testis is responsible for pulsatile retinoic acid signaling in spermatogenesis [72].

#### Cellular retinoic acid-binding proteins

Cellular retinoic acid-binding protein (CRABP) family are small cytosolic proteins that specifically bind RA in different tissues [73], and help with the solubilization of their hydrophobic ligands. CRABP I and II are exclusively intracellular proteins [74], and these RA-binding proteins play important roles to control the actual level of intracellular RA [75]. Previous studies have revealed that the expression pattern of CRABP1 and CRABP2 has been conserved between rat and mouse testis [60, 76, 77]. In the mouse testis, *CRABP1,* which is thought to target RA for degradation, was expressed only in the spermatogonia, but not in other germ cells and somatic cells [60]. It has been revealed that CRABP-I is exclusively localized in the cytoplasm of embryonic gonocytes and spermatogonia of the post-natal and adult testis but not in Sertoli cells [76, 77]. The exclusive expression of CRABP-I in the cytoplasm of gonocytes and spermatogonia indicates the possible role of CRABP-I in the degradation of RA in these actively dividing germ cells, and thereby preventing activation of retinoic acid receptors in spermatogonia [74, 77]. Surprisingly, a recent study results of genes differentially expressed using microarray analysis during the onset of puberty in fish showed a down-regulation of cytoplasmic RA-binding protein (*Crabp1*) [50].

It has been revealed that CRABP2 contributes in transfer of RA in the nuclear and hence is considerated as a molecular mediator for the biological effects of RA. Whereas CRABP1 has possible role in cytoplasmic degradation of RA via the cytochrome P450 family 26 (CYP26) enzymes. The results of a recent study by Nourashrafeddin et al. were showed a significant increase and decrease in expression of CRABP2 and CRABP1 expression after 11 days of hormone stimulation, respectively. Based on this results they reported that CRABP1 might be responsible for degradation of RA and maintenance of undifferentiated spermatogonia and CRABP2 might be responsible for activation of RA signaling in the Sertoli cells and initiation of spermatogonia differentiation [21].

The expression of Crabp-II, which is considered to promote RA signaling, was detected in only Sertoli and Leydig cells within the rat fetus testis [74, 77]. *Crabp-II* mRNA was detected at high levels in postnatal days in the rat testis [77]. The specific expression pattern of CRABP-II in prepubertal Sertoli cells which correlates with the developmental timing of Sertoli cell proliferation led Zheng et al., [77] to propose that CRABP-II is involved in the RA-dependent regulation of Sertoli cell proliferation . The specific expression pattern of Crabp-II in Sertoli cells also led Zheng et al., [77] to suggest that Sertoli cells might be the site of RA synthesis within the seminiferous tubules of the testis. Moreover, CRABP-II, but not CRABP-I, is essential to direct channeling of RA to the RA receptor-α (RARα) in the nucleus [78, 79], and it acts as a molecular mediator of RA activity [80]. All together these findings possibly imply that CRABP-I plays an important role in inactivation of RA activity, while CRABP-II promotes the nuclear transfer of RA, and hence is important for biological effects of RA.

The pattern of CRABP-II protein expression correlates with an increase in progesterone production in rats [77]. It has been revealed that over-expression of CRABP-II was involved in human germ cell tumor differentiation [81]. The expression of CRABP-II has up-regulated after 48 and 96 h of LH and FSH stimulation of juvenile monkey testis [20]. A recent study has demonstrated that the elevated levels of cytoplasmic CRABP-I are associated with RA resistance, while increased levels of CRABP-II are associated with sensitivity to RA activity [82]. The expression of CRABP-I inhibits RA signaling by degrading RA in the cytoplasm; therefor, these findings led Liu et al., to propose cellular RA-binding proteins as a biomarker for predicting cell response to RA [82]. Taken together, CRABP-I might be responsible for degradation of RA within undifferentiated spermatogonia and thereby prevents spermatogonia entering differentiation pathway in the juvenile monkey testis, whereas, up-regulation of CRABP-II could be responsible for the activation of RA signaling in Sertoli cells within seminiferous tubules of the monkey testis and as a result provides paracrine stimulation necessary for the initiation of spermatogonia differentiation and their meiotic entry.

#### Retinoic acid receptors

Within target cells, the effects of RA are mediated by the nuclear receptors for retinoic acid (RAR) and retinoid X (RXR). RAR and RXR are DNA-binding and transcription-modulating proteins involved in molecular mechanisms of the transcriptional responses in target genes. RA interacts with RAR/RXR heterodimer and binds to RA response elements (RAREs) in target genes and regulates the gene transcription [83]. In testis, both Sertoli and germ cells respond to RA via the RAR/RXR receptors. All isoforms of retinoic and retinoid receptors (RARA, RARB and RAR)) are expressed in the Sertoli and germ cells. It has been shown that RARα is responsible for the colonization and proliferation of germ cells in the basal area of the seminiferous tubules [13]. In Sertoli cells, RARα is responsible for differentiation of Sertoli cells [84]. RARα is necessary for the progression of germ cells through meiosis [13]. It has been demonstrated that the biological effect of RA is mediated through RARα in the germ cells [85]. In adult mouse testes, RARα is primarily localized in the nuclei of Sertoli cell and germ cells including spermatogonia and spermatocytes [60]. RARα plays a critical role in the regulation of germ cell development during spermatogenesis [9, 86]. It has been revealed that RARα-null mice are infertile [87]. While, RARβ null mice did not show any male infertility phenotypes [88]. RARγ is localized in the nuclei of undifferentiated Type A spermatogonia [60]. Although the RARγ-null male mice are fertile, they display an altered spermatogonial differentiation [89, 90]. Of retinoid X receptors, only RXRβ is critical for normal spermatogenesis [91]. The male RXRβ-null mice exhibit a delay in spermatid release from the seminiferous epithelium, the accumulation of cholesterol esters, and ultimately testis degeneration [91]. FSH signaling may explain how differentiating cells respond to RA activity. It has been demonstrated that FSH can stimulate Sertoli cell mitosis before puberty via controlling RARα. An increase in FSH levels during puberty stimulates RARα shuttling to the nucleus which is important for the differentiation of Sertoli cells [85, 92]. Spermatogonial stem cells containing RARs could maybe differentiate in response to RA but the expression of CYP26 results to degradation of RA and thereby prevent Spermatogonial differentiation.

#### Stimulated by Retinoic Acid 8 (Stra8)

Stra8 is considered as a marker for the action of RA on germ cells and its function is inevitable to initiate the transition between mitosis and meiosis [10, 15, 93, 94]. The exact function of STRA8 is not clear. It can shuttle between the nucleus and cytoplasm and bind to DNA [95]. STRA8 is expressed mainly in spermatogonia in accordance with its role in initiation of meiosis [96]. Activation of Stra8 expression in embryonic male germ cells induces the synthesis of downstream molecular markers of meiosis [11]. RA induces Stra8 in neonate testicular germ cells and causes meiotic initiation [12, 94]. STRA8 is only expressed in the differentiating spermatogonial population and required for their meiotic entry [15]. STRA8 protein accumulates in differentiating spermatogonia and preleptotene spermatocytes at stage VIII–IX of the cycle of the seminiferous epithelium [12]. A Study of Stra8 gene deletion revealed that the transition of Aal to A1 spermatogonia is blocked and germ cells fail to enter to meiosis. Therefore, only undifferentiated spermatogonia are present in the mouse testis [97].

The expression pattern of Stra8 in primate testicular cells does not appear to be as the same as in rodents. Recent studies have demonstrated no significant change in STRA8 expression after gonadotropins stimulation in Rhesus monkey testis [20, 72]. No expression of STRA8 was detected in the human testicular cells [56, 96]. However, STRA8 was weakly detected in the nuclei of small subset of differentiating B spermatogonia and spermatocytes in the human adult testes [96]. The expression of genes involved in RA signaling within testicular cells is summerized in Table 1.

**Table 1:** The expression of genes involved in RA activity within various testicular tissues/cells

Gene	Species	Stage of development	Cell expression	Conclusion
**RDH10**	Monkey	Juvenile/Adult	Spermatogonia	Sertoli cells may be the major source of Retinal in mouse testicular tissue
	Mouse	Juvenile	Spermatogonia/Sertoli cells	
**RDH11**	Mouse	Adult	Pachytene spermatocytes	The expression of RDH11 is highly stage-specific in sperm differentiation
**ALDH1A1**	Monkey	Juvenile/Adult	Sertoli cells	
	Human	Adult	Sertoli cells/peritubular myoid cells	The expression of ALDH1A1 is the major contributor to atRA formation
	Mouse	Postnatal/Adult	Sertoli cells/Leydig cells	The expression of Aldh1a1 in Sertoli cell precursors may play important functions in the differentiation of Sertoli cell precursors and primitive spermatogonia
**ALDH1A2**	Human	Adult	Undifferentiated spermatogonia/pachytene spermatocytes/spermatids/peritubular myoid cells	The expression of ALDH1A2 was predicted to be the predominant enzyme forming atRA in the testis
	Mouse	Postnatal	Pachytene/preleptotene spermatocyte	Aldh1a2 expression increased 7 and 8 days after the Aal–A1 transition ➔ the necessity of the endogenous RA in male germ cells is responsible for meiotic initiation
		Adult	Pachytene spermatocyte/Round spermatids	Aldh1a2 may modulate local RA concentrations
	Fish		Myoid and interstitial cells	Aldh1a2 is needed for meiotic initiation.
**ALDH1A3**	Human	Adult	Spermatogonia, Pachytene spermatocyte, Sertoli cells	
	Mouse	Adult	Sertoli and Lydig cell	Spermatogenesis is not altered in Aldh1a3-null mutants
**CRABP1**	Monkey	Juvenile testis	undifferentiated spermatogonia	Expression of CRABP1 was supressed after 11 days of gonadotropin treatment in juvenile testis
	Mouse	Developing testis/Adult	A and B spermatogonia	Spermatogenesis in not altered in Crabp1-null mutant
	Rat	Embryonic testis	gonocytes in earlier stages and spermatogonia later	CRABP1 protect these cells from the effects of RA
		Adult	A and B spermatogonia but not Sertoli cells	High CRABP levels in male germ cells reflect important role of retinoic acid in differentiation of these cells
**CRABP2**	Monkey	juvenile testis	Sertoli cells	Expression of CRABP2 was stimulated after 11 days of gonadotropin treatment in juvenile testis
	Mouse	Developing testis/adult	Not detected	spermatogenesis in not altered in Crabp2-null mutant
	Rat	Embryonic testis/Postnatal	Sertoli cells	CRABP-II is involved in RA-dependent regulation of Sertoli cell proliferation.
**STRA6**	Mouse	Adult	Sertoli cells	Stra6 was expressed in the same tubules as those expressing Stra8
**STRA8**	Human	Adult	B-spermatogonia, spermatocyte, spermatid, Leydig cells	
	Mouse	Postnatal	A and B spermatogonia, preleptotene spermatocyte	Stra8 expression is expressed in differentiating spermatogonia.
**CYP26B1**	Monkey	Juvenile	undifferentiated spermatogonia	CYP26B1 expressed in premieotic and pachytene sspermatocytes in a stage specific manner
		Adult	preleptotene, zygotene and pachetene spermatocytes	
	Mouse	Postnatal	germ cells (strong signal), Sertoli cells, peritubular myoepithelial cells	
		Adult	peritubular myoid cells	
**RARa**	Mouse	Postnatal/Adult	Sertoli cells, spermatogonia	RARA Functions in Sertoli Cells to Promote the Survival and Development of Early Meiotic Prophase Spermatocyte.
	Rat	Postnatal	Sertoli cells, early and late spermatocyte, elongating spermatid	The highest level of transcripts occurring in round spermatids at stage VIII of the spermatogenic cycle. RARa may be necessary for Sertoli cell differentiation
**RARb**	Mouse	Adult	steps 7 and 8 round spermatid	Colocalized with RXRa
	Rat	Postnatal	Sertoli cells, spermatogonia, early meiotic and pachytene spermatocytes	
**RARg**	Mouse	Postnatal/Adult	A spermatogonia, pachytene spermatocyte	
	Rat	Postnatal	Sertoli cells and Leydig cells	

#### Blood-testis barrier

**Fig. 2:**
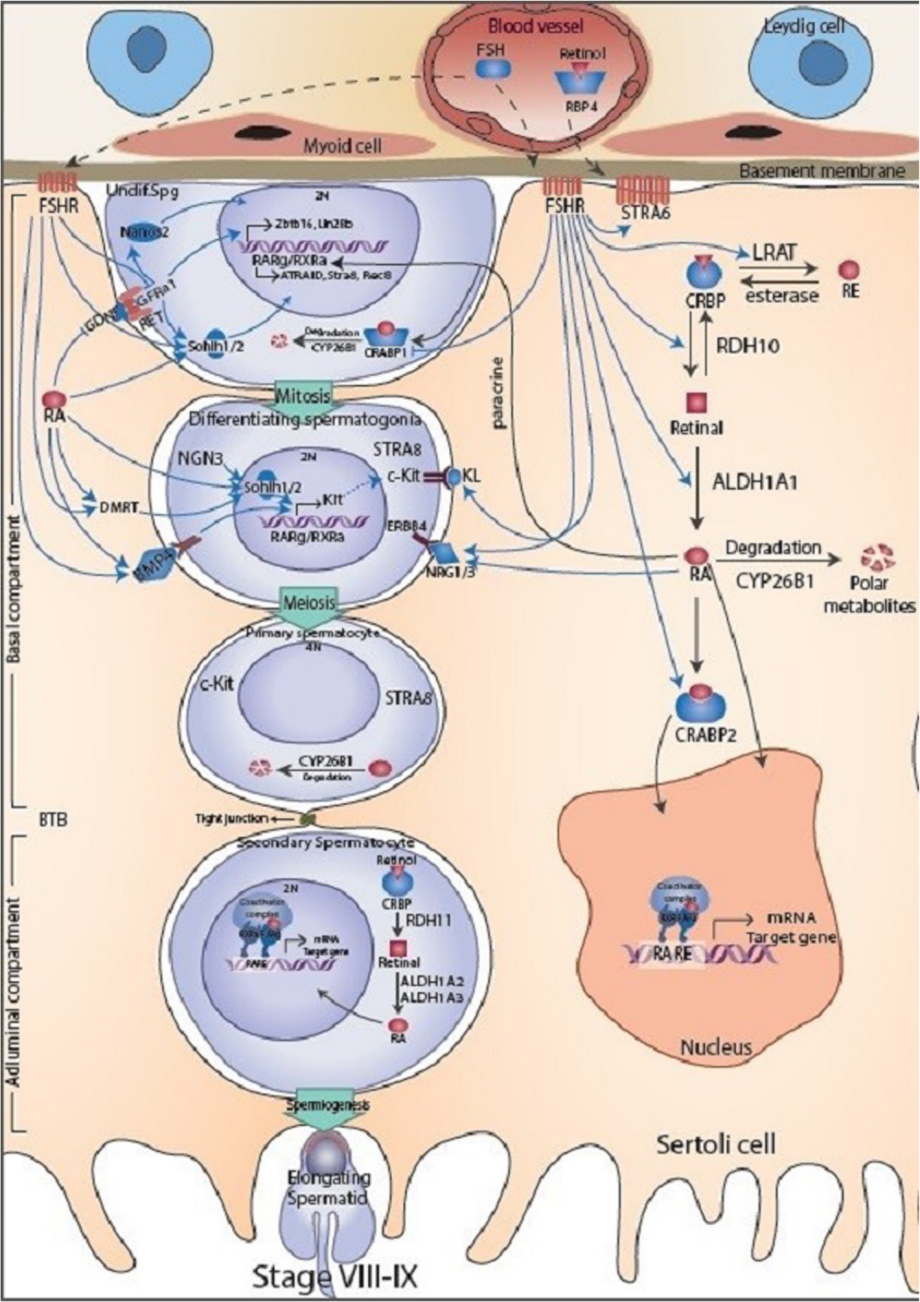
The possible paracrine mechanisms controlling differentiation of spermatogonia in adult testis. The spermatogenic cells arise from differentiation of undifferentiated type A spermatogonia (2n). The Ap spermatogonia can divide to form an Ap and a differentiatimg type B spermatogonia (2n) through the cycle of the seminiferous epithelium stages VIII to IX. B spermatogonia divide randomly and differentiate to form primary (4n) and secondary spermatocytes (2n) through meiotic divisions. The secondary spermatocytes proceed through the rest of meiosis to form elongating spermatids (n). Differentiation of spermatogonia occurs during stages of VII-VIII when the RA concentration is at the highest levels. FSH is necessary for early-stage of spermatogonia differentiation and their meiotic entry. FSH induces spermatogonia differentiation and their meiotic entry thorough controlling RA signaling and several growth factors acting on the early stages of mammalian spermatogenesis. In one hand, FSH probably induces the activation of RA signaling pathway notably through increase Rdh10, Aldh1a1, Crabp2 in Sertoli cells and decrease Crabp1 in spermatogonia and thereby provide a robust paracrine factor necessary for induction of spermatogonia differentiation. In another hand, FSH induces components of signaling pathways regulated by RA signaling such as Sohlh1/2, Kit ligand, DMRT, BMP4 and NRGs along with transcription factors that are important for proliferation and differentiation of spermatogonia and their meiotic entry. Exogenous and endogenous factors are represented in Sertoli and germ cells, respectively. Black arrows refer to retinol metabolism and RA signaling pathway. The succession of the various types of germ cells during the stages of VIII-IX seminiferous epithelium is represented between Sertoli cells: AS, undifferentiated type A spermatogonia; BS, differentiating type B spermatogonia; PS, pre-leptotene or primary spermatocytes; SS, secondary spermatocytes; ES, elongating spermatocytes. FSH**,** Follicle Stimulating Hormone, FSHR, Follicle Stimulating Hormone receptor; KL, Kit ligand ligand; DMRT, Doublesex And Mab-3 Related Transcription Factor; BMP4, Bone Morphogenetic Protein 4; NRG, Neuregulin; BTB, blood testis barrier

The blood-testis barrier (BTB) is one of the tightest blood-tissue barriers in the mammalian cells. It divides the seminiferous epithelium into the basal and the adluminal parts (Fig. 2). Spermatogonial renewal and differentiation up to the preleptotene spermatocyte stage take place outside of the BTB in the basal compartment of the epithelium, but meiosis, spermiogenesis, and spermiation all take place in a microenvironment behind the BTB in the adluminal part. It has been shown that the expression of genes associated with the establishment of blood-testis barrier were up-regulated following gonadotropin stimulation for 48 h or 96 h in juvenile monkey testis [20]. For instance, claudin 11 (CLDN11), a major component of the blood-testis barrier, was overexpressed due to gonadotropin stimulation.

### Competency of germ cells to enter meiosis: role of FSH

#### Control of spermatogenesis via spermatogonia stem cell niche

Commitment of undifferentiated A spermatogonia to meiosis occurs in the postnatal testis. Spermatogonia undergoes cycles of self-renewal and differentiation within the seminiferous epithelium. Interactions between Sertoli cells and germ cells are essential for the normal progression of spermatogenesis [98]. FSH binds to FSHR expressed by Sertoli cells in the seminiferous tubules, and leads to the activation of genes needed for regulation of spermatogenesis. Differentiation of spermatogonia and their entry to meiosis also is controlled through several growth factors acting on the early stages of mammalian spermatogenesis [99]. Sertoli cells produce growth factors that stimulate self-renewal and differentiation of spermatogonia, including Activin A, Glial cell-derived neurotrophic factor (GDNF), Kit ligand (KL), Bone morphogenetic protein 4 (BMP4), and Neuregulins (NRGs).

Growth factors are important for maintaining stem cell self-renewal and normal spermatogenesis in the postnatal testis. GDNF is an essential mediator of the influence of pituitary gland on the spermatogenic process. GDNF is produced by Sertoli cells and plays important roles in survival, self-renewal and proliferation of undifferentiated Type A spermatogonia [100]. GDNF positively stimulates spermatogonia self-renewal but negatively inhibits their differentiation. Undifferentiated spermatogonia express c-Ret, a high affinity receptor for GDNF, as well as GDNF family receptor α1 (GFRα1). Both c-Ret and GFRα1 are required for the binding of GDNF produced by Sertoli [101]. Ret proto-oncogene and GFRα1 is expressed by undifferentiated spermatogonia in the postnatal mouse testis [102, 103]. Previous studies have demonstrated that spermatogonia are absent in GDNF gene/receptor knocked out testis [104, 105]. Therefore; GDNF-GFRa1 pathway plays a major role in the regulation of self-renewal and maintenance of undifferentiated spermatogonia in mammals. The transcript of GFRα1 was preferentially expressed on a population of undifferentiated A-spermatogonia with a high stemness behavior and preferentially expressed NANOS2, a RNA-binding protein that promote self-renewal of spermatogonia [106]. It has been demonstrated that the balance between self-renewal and differentiation of spermatogonia during early stages of the spermatogenesis process is under paracrine regulation by FSH via the control of GFRα1 expression [106]. A recent study has shown that the expression of GFRα1-NANOS2 signaling was repressed after 48 and 96 h of LH and FSH stimulation of juvenile monkey testis [20]. The commitment of undifferentiated spermatogonia to differentiation pathway and meiosis via RA happens within stages VII–IX. It has been reavaled that the expression levels of GDNF by Sertoli cells was highest at stages XII–I and lowest at stages VII–VIII [103]. The stage-specific expression of GDNF is critical in proliferation of undifferentiated spermatogonia at stages XII–I [107]. This findings lead us to suggest that FSH increase differentiation of spermatogonia via activation of RA signaling in Sertoli cells and also it control self-renewal and differentiation of spermatogonia via regulation of GFRα1expression in undifferentiated spermatogonia in a stage specific manner.

Differentiating A1 spermatogonia undergo a number of proliferative cycles to form the A_2_–A_4_, intermediate, and B spermatogonia. Differentiating A1 spermatogonia expresses Kit, a gene that encodes a tyrosine kinase receptor for KL expressed by Sertoli cells [108, 109]. GFRα1 expressing spermatogonia can be considered as a stem cell pool within the testis. Upon down-regulation of GFRα1, more differentiated states expressing NGN3 or Kit, appear in the seminiferous testis [110, 111].

#### Control of spermatogenesis via regulation of kit expression

Previous studies demonstrated that Kit expression plays an essential role in the control of spermatogonia proliferation and their meiotic entry. Kit has been considered as a molecular marker of entering the differentiation pathway and the meiotic phase of spermatogenesis. Therefore, understanding the mechanisms which regulate Kit expression will increase our knowledge on how the initial phases of spermatogenesis are regulated.

Kit ligand (KL) is produced by the somatic cells that regulate survival and differentiation of germ cells. KL is considered as an essential mediator of the influence of gonadotropin on the spermatogenic process. It has been demonstrated that KL expression is induced by FSH in pre-pubertal mouse Sertoli cells culture [112, 113] through an increase in cAMP levels [114]. It has been reported that c-Kit expressed and localized in spermatogonia within the adult human testis in a stage-specific manner [115]. It has been shown that induction of KL occurs in a specific stage manner within adult seminiferous tubules which are sensitive to FSH stimulation. In the adult mouse testis, stages of seminiferous epithelium which are sensitive to FSH stimulation show higher levels of the Kit [116]. KL/Kit interactions are essential for proliferation of differentiating type A spermatogonia, transition from type B spermatogonia to pre-leptotene spermatocytes and their meiotic entry [117]. Upon onset of meiosis Kit is down-regulated and its mRNA and relevant protein cannot be detected in pachytene spermatocytes.

Spermatogenesis and Oogenesis b-Helix-Loop-Helix (HLH) transcription factors, SOHLH1 and SOHLH2, specifically are expressed in spermatogonial germ cells, and are critical for the differentiation of spermatogonia. Deletion of each Sohlh1 and Sohlh2 transcription factors lead the elimination of Kit-expressing spermatogonia in the testis [118, 119], suggesting SOHLH1 and SOHLH2 positively regulate the expression of KIT transcripts in germ cells and involve in the differentiation of spermatogonia [111, 119, 120]. It has been found a strong correlation among Sohlh1, Sohlh2 and Kit expression in postnatal spermatogonia [111, 120]. Sohlh1 expression was maximal in Kit positive cells, whereas Sohlh2 was found to enrich in undifferentiated spermatogonia. It has been revealed that Sohlh1 interacts with discrete bHLH binding site containing regions within the Kit promoter in spermatogonia [119, 120]. A recent study conducted by Plant’s lab has shown that Sohlh1 shuttling to the nucleus increases c-Kit expression during monkey puberty [111].

On the other hand, RA is considered as a positive regulator of Kit expression and spermatogonial differentiation [114, 120]. It had been found that RA rapidly and strongly induces Kit expression in differentiating spermatogonia [120]. Interestingly, expression of Sohlh1 was increased in differentiating pre-meiotic germ cells by treatment with all-trans RA [120], suggesting that RA stimulates Kit expression and spermatogonia meiotic entry. Recent study has determined that RA induces the expression of KIT through the PI3K/AKT/mTOR signaling pathway [121]. It has been suggested that RA stimulates PI3K/AKT/mTOR kinase signaling in differentiating spermatogonia, and transcriptionally activates genes required for meiosis (e.g., *Stra8* and *Rec8*). Moreover, RA enhances the translational of spermatogonial differentiation markers (e.g., *Kit*, *Sohlh1*, and *Sohlh2*) through activation of kinase signaling [122]. These results might be another evidence that FSH correlates with RA to drive KIT up-regulation and thereby govern spermatogonia into differentiation pathway.

BMP4, a ligand for the transforming growth factor-β (TGFβ) superfamily, is an essential mediator of the influence of FSH on the spermatogenic process. It has been demonstrated that BMP4 regulates proliferation of mouse spermatogonial stem cells and mediates their differentiation in Kit expressing cells [123]. BMP4 is expressed in prepuberal Sertoli cells but down-regulated at puberty. It has been found that cAMP analogues down-regulate BMP4 levels in prepuberal and pubertal Sertoli cells while RA strongly up-regulates BMP4 levels at both ages, suggesting a negative control from the hypothalamic- pituitary axis [123]. Recent study has revealed that the expression of BMP4 is down-regulated after 48 h of LH and FSH stimulation [20].

It has been found that Neuregulins (NRGs), a member of the epidermal growth factor family, are expressed by Sertoli cells in testicular tissue. Neuregulin’s receptor, ERBB4, has been shown to be localized on germ cells [124, 125]. The analyses of NRG1 null mice have demonstrated that RA and FSH act on Sertoli cells to promote the expression of NRG1 and NRG3 and thereby induce spermatogonia proliferation and their meiotic initiation [125]. However, the molecular mechanisms by which RA/FSH promotes NRG1/3 gene expression in Sertoli cells and how NRG1/3 stimulates the expression of *Stra8* requires further elucidations.

Spermatogenesis is regulated by Inhibin-α and Activin, a member of the TGFβ superfamily, secreted by Sertoli cells [126]. Inhibin-α negatively regulates the FSH secretion from the pituitary gland [127]. Both RA and FSH promote the secretion of transferrin, androgen-binding protein (ABP) and inhibin-α [128, 129].

Several transcription factors important for the pluripotency, proliferation and maintenance of undifferentiated spermatogonia have been described, including POU5F1, ZBTB16, NANOG and LIN28 [20, 130]. The expression of transcriptional repressor ZBTB16 is considered as a marker for undifferentiated spermatogonia [20, 131]. ZBTB16 is coexpressed with pluripotency gene marker POU5F1 on undifferentiated spermatogomia and regulates their epigenetic state [131]. Pluripotency transcription factors POU5F1 and NANOG are necessary for competency of spermatogonia to enter meiosis [132].

Promyelocytic Leukemia Zinc Finger (PLZF), also known as ZBTB16, controls expression of Kit specifically in spermatogonia. PLZF is a DNA sequence-specific transcriptional repressor expressed in undifferentiated spermatogonia but not in Kit-positive spermatogonia. It has been found that PLZF directly represses Kit in undifferentiated spermatogonia [133]. Deletion of PLZF in KO mice induced expression of genes controlling the switch between spermatogonial self-renewal and differentiation causing exhaustion of these cell populations [134].

Doublesex and mab-3 related transcription factor (DMRT1) is an important intrinsic factor in the decision of spermatogonia to commit into the differentiation pathway. DMRT1 is expressed by both germ and Sertoli cells [135]. Previous study has demonstrated that DMRT1 directly prevents transcription of STRA8 in differentiating spermatogonia and apparently opposes the action of RA [135]. Germ cell-specific deletion of DMRT1 in adult spermatogenesis results in the ability of spermatogonia to induce Stra8 and enter meiosis. It has been shown that deletion of DMRT1 in spermatogonia prohibits the differentiation of spermatogonia and their meiotic entry [135]. DMRT1 promotes spermatogonial differentiation via activation of SOHLH1, a spermatogonial differentiation factor [135]. In the adult testes, DMRT1 was mostly expressed in spermatogonia, except in the A-dark type [96]. It has been found that the expression of DMRT1 was up-regulated after 48 h of LH and FSH stimulation [20]. A robust down regulation of KIT expression in spermatogonia was seen in DMRT1 conditional KO mice testes [135].

Inhibition of the factors needed for maintaining the undifferentiated state governs commitment of undifferentiated spermatogonia to the pathway of differentiation. Gene expression analysis revealed that the expression of LIN28 was down-regulated in the testis of juvenile rhesus monkeys after gonadotropin treatment [20]. Differentiation of spermatogonia via RA leads to the suppression of LIN28, and as a result, the Mirlet7 family are induced and further down-regulated genes associated with self-renewal of spermatogonia [136].

## Conclusion

Spermatogenesis is a complex process of sperm production that is controlled by interactions between spermatogenic and somatic cells. This process in mammals initiates at puberty with differentiation of undifferentiated spermatogonia and their meiotic entry [1]. The commitment of undifferentiated spermatogomia to differentiating spermatogonia and normal spermatogenesis requires the action of gonadotropins FSH and LH. At prepubertal development phase in the juvenile testis and/or in the absence of gonadotropins only Sertoli cells and undifferentiated germ cells are present in the seminiferous tubules [3, 4] (Fig. 1). Spermatogenic cells are in close contact with Sertoli cells which provide structural support and paracrine factors to regulate spermatogonia self-renewal and differentiation. In higher primate, spermatogenic cells arise from differentiation of undifferentiated type A spermatogonia (2n). The Ap spermatogonia can divide to form an Ap and a differentiating type B spermatogonia (2n) through the cycle of the seminiferous epithelium stages VIII to IX. B spermatogonia divide randomly and differentiate to form primary (4n) and secondary spermatocytes (2n) through meiotic divisions. The secondary spermatocytes proceed through the rest of meiosis to form elongating spermatids (n) (Fig. 2). The identification of positive and negative regulators of early stages of spermatogenesis processes is important for understanding how spermatogenesis can be controlled and influenced by extrinsic and intrinsic regulators.

On the other hand, it has been demonstrated that vitamin A deficient (VAD) animals are infertile and only undifferentiated spermatogonia and premeiotic germ cells are present in the seminiferous tubules [8]. Recent studies have demonstrated that RA, the metabolic active form of vitamin A, is responsible for the cyclic differentiation of germ cells and the continual production of sperm in the adult testis [7]. As the expression of Cyp26b1 is expressed by the peritubular myoid cells, cells within the seminiferous epithelium are considered to synthesize and control RA concentrations [60]. It has been suggested that both Sertoli and germ cells have the ability to generate and degrade RA [137]. Moreover, it has been shown that RA is primarily synthesized by the Sertoli cells within the testis [138]. In Sertoli cells, retinol is oxidized to retinal by RDH10 and then to RA by RALDH1a1 (Figs. 1 and 2). However, it looks that the amount of RA produced by Sertoli cells within juvenile testis is not robust to initiate spermatogonia differentiation possibly due to the low amount of CRABP2 which is considered for activating of RA signaling in Sertoli cells as well as the high amount of CRABP1 which is considered for degrading of RA signaling within spermatogonia. Therefore, in juvenile testis, RA might be responsible for proliferating and self-renewing of undifferentiated spermatogonia in a relatively gonadotropin-independent manner.

Recent studies have increased our knowledge of the early molecular and endocrine events that trigger pubertal development and the onset of spermatogenesis. It has been demonstrated that FSH controls Sertoli cell’s function and orchestrates mitotic-to-meiotic progression during the first wave of murine spermatogenic development [139]. Gene deletion studies demonstrate the requirement of FSH for early-stage of spermatogonia differentiation and their meiotic entry [32–34]. FSH regulates the initiation of spermatogonial differentiation and their meiotic entry by controlling the expression of growth factors which are essential for proliferation and differentiation of spermatogonia. A synergistic effect of FSH and vitamin A on differentiation of the testicular germ cell was observed in the adult cryptorchid testis, which only consists of undifferentiated spermatogonia and Sertoli cells [140]. Moreover, it has been revealed that the basal serum FSH and LH levels in VAD rats is higher than controls [141]. Interestingly, recent gene expression studies showed that several genes involved in the RA signaling pathway including RBP4, LRAT, Rdh10, Aldh1a2, Cyp26a1, Crabp1/2 as well as RARα are influenced by changing in gonadotropin levels [20, 50, 72].

RA signaling plays an important role in the induction of germ cell differentiation and meiotic entry. In mice, RA synthesized by Sertoli cells initiates the first round of spermatogenesis [58]. In the first round of spermatogenesis, RA synthesized by the Sertoli cells in a paracrine manner acts on spermatogonial cells to initiate the A to A1 transition; however, after that RA synthesized in spermatocytes stimulates the cycles of spermatogenesis [7]. It has been found that synchronous spermatogenesis in neonatal mice (2 dpp), but not after initiation of meiosis (8 dpp), can be induced by treating vitamin A sufficient males with RA [142]. The treatment of neonatal males at different ages with exogenous RA has revealed that although RA is sufficient to induce differentiation of spermatogonial at 4 dpp and earlier, but it failed to cause spermatogonial differentiation after the appearance of meiotic preleptotene spermatocytes [143].

Studies on the regulatory effect of gonadotropin on RA activity during meiotic progression has been revealed that availability of RA is regulated by coordinate synthesis and degradation. This is a critical determinant of several spermatogenic stages, including FSH-regulated mitotic stage (PND5) and androgen-dependent meiotic stage (PND10) [139]. It has been found that meiotic spermatogonial development stage (PND5) in murine testis coincides with elevated transcripts of RA synthesizing enzymes *Aldh1a1* and *Adh1,* and low expression of RA catabolic enzyme *Cyp26b1*. While the expression of *Aldh1a1/Adh1* down-regulated and *Cyp26b1* up-regulated during initial meiotic progression (PND10) which cause a transient reduction in RA availability during the first wave of meiotic progression (PND10) [139]. Moreover, study by Hazra et al., showed that the expression levels of Inhibin and Activin A receptor (*Acvr2b*) were highest during low *Cyp26b1* expression at PND5, and then reduced around the peak of *Cyp26b1* expression in wild type testes at PND10. They suggest that down-regulation of Activin A reduces its inhibitory effects on *Cyp26b1* and thereby allowing Cyp26b1-mediated regulation of early meiosis [139]. This evidence suggest that FSH probably induces the activation of RA signaling pathway through increase Aldh1a1 in Sertoli cells and decrease Cyp26b1 in spermatogonia and thereby provide a paracrine factor necessary for commitment of spermatogonia to differentiation pathway.

During puberty, FSH induces components of signaling pathways regulated by RA signaling such as GDNF, Sohlh1/2, KL, DMRT, BMP4 and NRGs along with transcription factors that are important for proliferation and differentiation of spermatogonia and their meiotic entry. GDNF-GFRa1 pathway plays a vital role in the regulation of self-renewal and maintenance of undifferentiated spermatogonia in mammals. GDNF expressed by Sertoli cells is responsible for transcriptional activation of Zbtb16 and Lin28B via GFRa1 signaling and thereby preventing spermatogonia to enter the differentiation pathway (Figs. 1 and 2). A recent study has demonstrated that undifferentiated GFRA1^+^ spermatogonia cannot respond to RA either by lack of requisite RARs or degradation by CYP26 enzymes in the spermatogonium (Fig. 1). Whereas, differentiating STRA8^+^ and KIT^+^ spermatogonia responds to RA either by expression of RAR or the absence of localized CYP26 degrading enzyme within the spermatogonium or adjacent Sertoli cells [122] (Fig. 2). It has been demonstrated that FSH and RA regulates the activity of GDNF and NOTHC signaling in Sertoli cells [112, 144, 145]. Interestingly, retinoids regulate the expression of GDNF messenger molecules in response to FSH [146–149] (Fig. 2). Moreover, it has been found that several genes involved in NOTCH signaling pathway are influenced by gonadotropin treatment [20]. The expression of JAG1, a component of NOTCH signaling pathway, is increased at stage VII–VIII [145, 150]. It had been shown that FSH and RA signaling changes in a stage-dependent manner [103, 151]. These finding lead us to suggest that FSH signaling may cause the periodical expression of genes involved in RA signaling and thereby possibly triggers the initiation of the seminiferous epithelial cycle in a stage specific manner (Table 2).

**Table 2:**
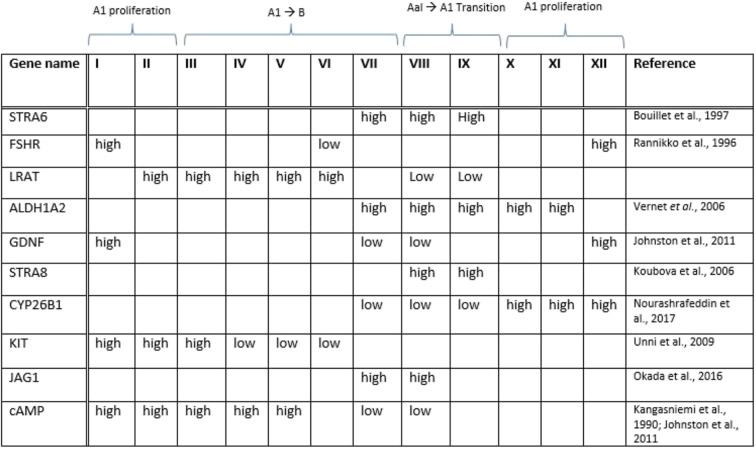
Differentiation of spermatogonia by RA signaling in adult testis is possibly controlled by FSH in a stage-specific process limited to stage VIII/IX tubules. The table illustrate a hypothetical pulse of RA based on the testicular response to FSH as described in the text. The cycle begins with the transition of undifferentiated spermatogonia (Aal) to form differentiating A1 and then B spermatogonia

In summary, the interactions between retinoids and FSH are complex. In one hand, FSH probably induces the activation of RA signaling pathway notably through increase Rdh10, Aldh1a1, Crabp2 in Sertoli cells and decrease Crabp1 in spermatogonia and thereby provide a robust paracrine factor necessary for induction of spermatogonia differentiation. In another hand, FSH induces components of signaling pathways regulated by RA signaling such as GDNF, Sohlh1/2, KL, DMRT, BMP4 and NRGs along with transcription factors that are important for proliferation and differentiation of spermatogonia and their meiotic entry. These investigations provide the novel finding that implicates gonadotropin FSH as key factors involved in the regulation of RA activity in the initiation of spermatogonia differentiation and spermatogenesis in the mammal testis. The overall expression pattern of RA signaling related genes indicates an important role of gonadotropins in regulating the spermatogonia renewal–differentiation switch presumably through regulation of CRABP1 and CRABP2 expression. According to all studies that demonstrate the interface between FSH and RA signaling, we suggest that RA may trigger spermatogonia differentiation and initiation of meiosis through regulation by FSH signaling in the seminiferous tubules within the testis. This is a novel finding on the mechanisms of gonadotropins to control spermatogenesis via RA, which is considered to be responsible for the cyclic differentiation of germ cells in the adult testis and the continual production of sperm. Therefore, to the best of our knowledge, this is the first time that the correlation between FSH and RA signaling in spermatogenesis is highlighted. However, the molecular mechanisms by which FSH control spermatogenesis via RA signaling need further investigations.

## Data Availability

All data generated or analyzed during this study are included in this published article.

## Abbreviations

RA: Retinoic Acid
TGFβ: Transforming Growth Factor-β
GDNF: Glial Cell-Derived Neurotrophic Factor
BMP4: Bone Morphogenetic Protein 4
FSH: Follicle-Stimulating Hormone
SOHLH1: Spermatogenesis and Oogenesis Specific Basic Helix-Loop-Helix 1
DMRT1: Doublesex and mab-3 related transcription factor
PLZF: Promyelocytic Leukemia Zinc Finger
LH: Luteinizing hormone
GnRH: Gonadotropin-Releasing Hormone
VAD: Vitamin A Deficient
CRABP2: Cellular Retinoic Acid Binding Protein 2
PGCs: Primordial Germ Cells
RBP: Retinol-Binding Protein
RDH10: Retinol Dehydrogenase 10
ALDH1A2: Aldehyde Dehydrogenase 1 Family Member A2
TSA: Ttrichostatin A
RAR: Receptors for Retinoic Acid
Stra8: Stimulated by retinoic acid 8
BTB: Blood-Testis Barrier
CLDN11: Claudin 11
GFRα1: GDNF family receptor α1
KL: Kit ligand
PND: Post-natal Day
